# Metabolic surgery versus usual care effects on diabetes remission: a systematic review and meta-analysis

**DOI:** 10.1186/s13098-023-01001-4

**Published:** 2023-02-24

**Authors:** Hyder Mirghani, Ibrahim Altedlawi Albalawi

**Affiliations:** 1 Department of Internal Medicine, Faculty of Medicine, University of Tabuk, PO Box 3378, Tabuk, 51941 Saudi Arabia; 2 Department of Surgery, Faculty of Medicine, University of Tabuk, Tabuk, Saudi Arabia

**Keywords:** Bariatric surgery, Metabolic surgery, Diabetes remission, Usual care, Lifestyles

## Abstract

**Background:**

Bariatric surgery is superior to usual care for diabetes remission. Previous meta-analyses were limited by pooling observational and randomized trials, using various definitions of diabetes remission, and not controlling for various diabetes medications. The current meta-analysis aimed to compare bariatric surgery and usual care regarding the same.

**Methods:**

We searched PubMed MEDLINE, Web of Science, SCOPUS, and Cochrane Library for relevant articles from the date of the first inception up to February 2023. The keywords diabetes remission, Bariatric surgery, metabolic surgery, lifestyles, usual care, GLIP-1 agonists, insulin use, gastric banding, biliopancreatic diversion, sleeve gastrectomy, and Roux-en-Y gastric bypass, were used. A datasheet was used to extract the relevant data.

**Results:**

Diabetes remission (complete and prolonged) was higher among bariatric surgeries compared to usual care, odd ratio, 0.06, 95 *CI*, 0.02–0.25 and 0.12, 95 *CI*, 0.02–0.72, respectively. bariatric surgery patients were younger, had higher HbA1c, odd ratio, − 3.13, 95 CI, − 3.71 to 2.54, and 0.25, 95 CI, 0.02–0.48, respectively, insulin use was higher, and glucagon-like peptide agonists use was lower among bariatric surgery patients, odd ratio, 0.49, 95% *CI*, 0.24–0.97, and 3.06, 95% *CI*, 1.44–6.53, respectively.

**Conclusion:**

Bariatric surgery was better than usual care in diabetes remission. Bariatric surgery patients were younger, had higher HbA1c, and received more insulin and lower GLP-1 agonists. No differences were evident regarding body mass index and the duration of diabetes. Further trials comparing the new anti-diabetic medications and different forms of bariatric surgery and controlling for the level of exercise and diet are recommended.

## Background

Diabetes mellitus is a chronic progressive disorder with a great burden on the patients, the healthcare system, and the community, according to International Diabetes Federation, 463 million adults were suffering from diabetes in the year 2019 with an annual death of 4.2 million globally. The global economic burden of diabetes is USD 760.3 billion in 2019 [1]. Diabetes mellitus is a progressive disease with high microvascular and macrovascular complications. In addition, the disease is associated with high mortality [2]. Diabetes mellitus and obesity are growing health burdens globally; they are usually associated (with diabesity) and put a high strain on the healthcare system.

Type 2 diabetes remission and reversal of metabolic abnormalities have been gaining momentum recently [3, 4]. Diabetes remission is defined as the glycated hemoglobin < 6.5% (48 mmol/mol) measured at least 3 months after stopping diabetes medications and is associated with reduced microvascular complications in particular among young age groups and those with lower comorbidities [5].

Diabetes mellitus remission can be induced with lifestyles (various dietary approaches including low calorie or low carbohydrate diet and various grades of physical activity), surgical intervention (bariatric surgery), and novel antidiabetic medications [6–8].

American College of Lifestyle Medicine's position statement is to target diabetes remission as the primary goal for patients with type 2 diabetes and use a therapeutic diet and exercise. Bariatric surgery showed similar results. However, lifestyles bear fewer side effects [9]. A recent review of meta-analysis stated that low-energy diets and formula meal replacement appear the most effective approaches, generally providing less energy than self-administered food-based diets with no support for any particular macronutrient profile or style [10].

Diabetes prevention through lifestyle modification backdated to the 1980s, evidence from a meta-analysis of randomized controlled trials showed the feasibility of sustained prevention of diabetes by reducing weight, physical activity, and Mediterranean diet [11]. World Health Organization (WHO) acknowledged diabetes reversal by caloric restriction [12], in addition, studies showed the effectiveness of bariatric surgery, carbohydrate restriction, or low-calorie diets [13].

It is stated that a 10% weight reduction is needed for meaningful health improvement. However, weight loss is difficult to achieve through lifestyle alone [14]. A large cohort published in the United Kingdom found that 5% weight loss is achievable in 14.3% and 12.5% of women and men, respectively [15]. Initial weight loss and dysglycemia can be obtained by different dietary approaches at the expense of constipation, alopecia, and cholelithiasis [16]. While cost might be a limiting factor for some diets [17]. Low carbohydrate diets for diabetes management is in use for more than two centuries and were found to be effective as an alternative to low-fat, low-calorie diets [18]. Previous studies showed weight reduction and diabetes remission through intensive lifestyle intervention [19, 20]. Other approaches for remission include novel hypoglycemic medications and bariatric surgery [3]. Gastrointestinal interventions intended for long-term weight loss have evolved since the fifties of the previous century. The new term metabolic surgery was developed to replace bariatric surgery given the concept of morbidity-related obesity surgeries. The term indicates improvement or even remission of metabolic disorders including type 2 diabetes [21]. The improvement in obesity-related metabolic disorders is mainly through sustainable weight loss and neuroendocrine mechanisms. Nearly a half million bariatric surgeries are currently performed annually worldwide [22]. There are many bariatric surgeries including sleeve gastrectomy, Roux-en-Y gastric bypass, and laparoscopic adjustable gastric banding. With the development of laparoscopic techniques, now laparoscopic sleeve gastrectomy (LSG) is gaining favor over other procedures [23].

Bariatric surgery was shown to induce sustainable diabetes remission between one to two-thirds depending on the surgical procedure [24].

Exercise plays a pivotal role in preventing and controlling DM since its effects include most vascular risk factors, with special effects on diabetes. Exercise is the best non-pharmacological therapy for the population in question [25–28].

A previous meta-analysis showed the beneficial effects of moderate-intensity exercise on type 2 diabetes risk (30% risk reduction). The same was observed with regular walking. In addition, the results remained robot after controlling for body mass index [29]. Literature on diabetes remission with bariatric surgery and usual diabetes care lacks. The available meta-analyses on comparisons are limited by pooling observational studies, using various definitions of diabetes remission, and including small studies. Therefore, we conducted this meta-analysis to compare usual care and bariatric surgery effects on diabetes remission and include randomized controlled trials with the most recent definition of diabetes remission.

## Materials and methods

### Eligibility criteria according to PICOS

Studies were included if they were randomized controlled trials on humans and compared bariatric surgery and usual diabetes care regarding diabetes remission. Retrospective studies, prospective cohorts, cross-sectional studies, case control, and case series were excluded.

### Outcome measures

The outcome measures were:

- Diabetes remission following bariatric surgery and usual care.

### Diabetes remission

Diabetes remission is defined as the achievement of HbA1c of < 6.5 without diabetes medication for three months or longer. Prolonged remission is the maintenance of the same for one year, and permanent remission is HbA1c < 6.5 for five years or more. A glycated hemoglobin of < 6.5 estimated from the mean blood glucose using continuous glucose measurement and fasting blood glucose of < 126 mg/dl are acceptable [4].

### Literature search and data extraction

**Fig. 1:**
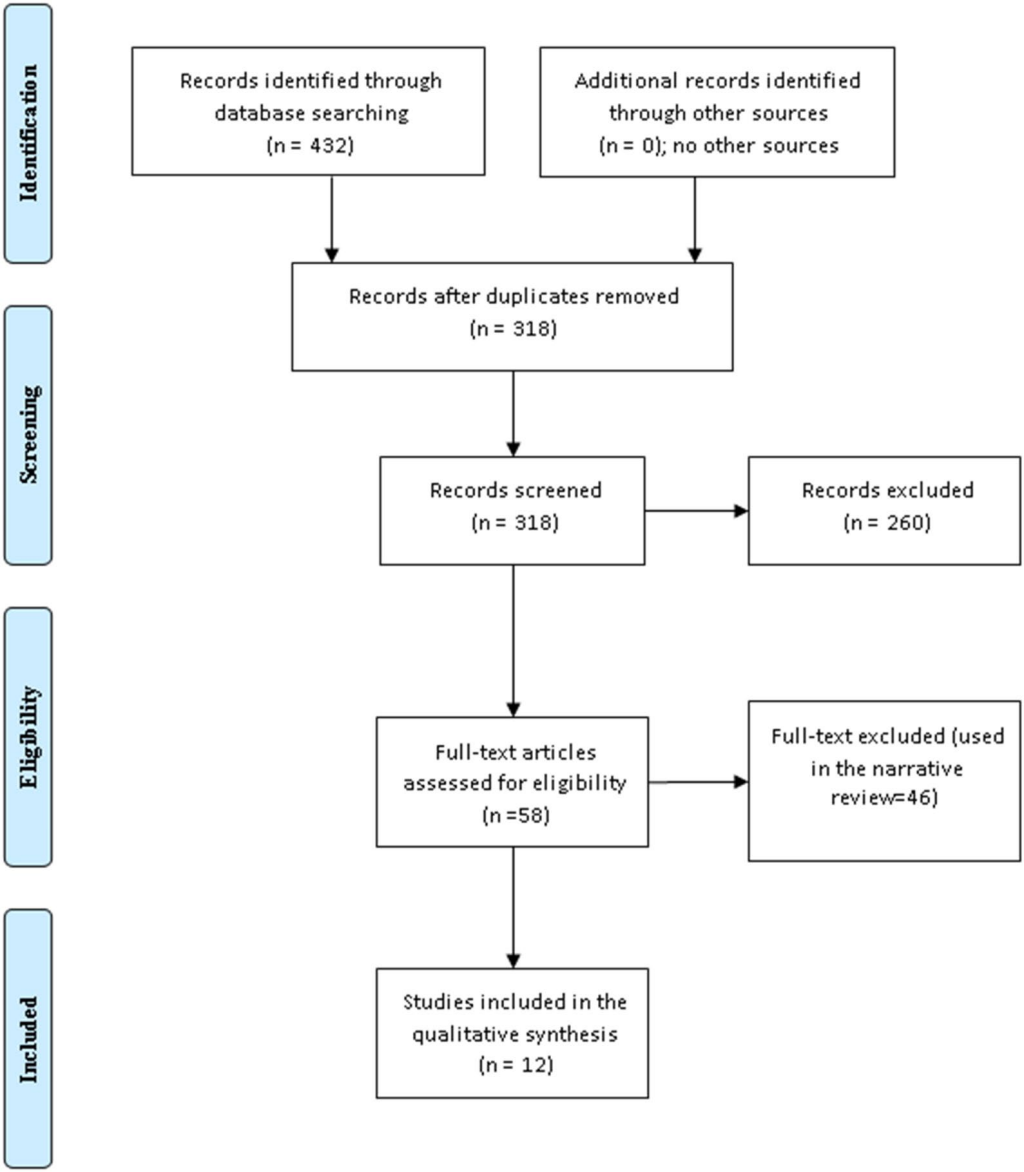
Randomized controlled trials comparing different types of bariatric surgeries and medical treatments (the PRISMA chart)

**Fig. 2:**
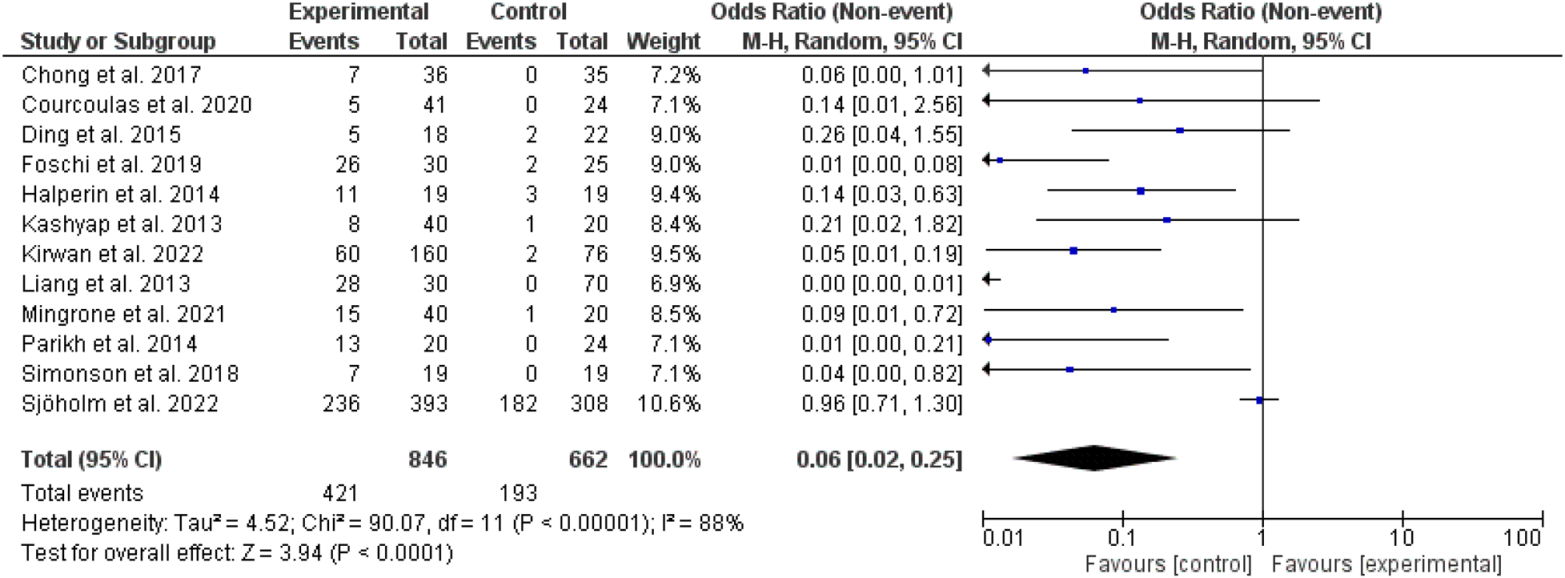
Diabetes remission among different types of bariatric surgeries and medical treatments

**Fig. 3:**
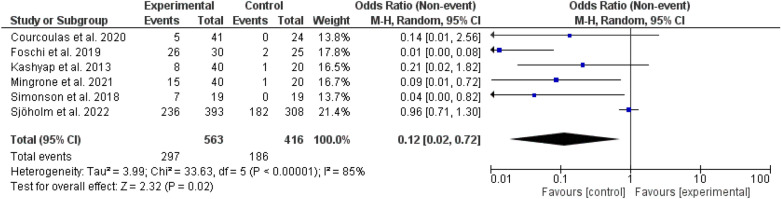
Complete and prolonged diabetes remission among different types of bariatric surgeries and medical treatments

**Fig. 4:**
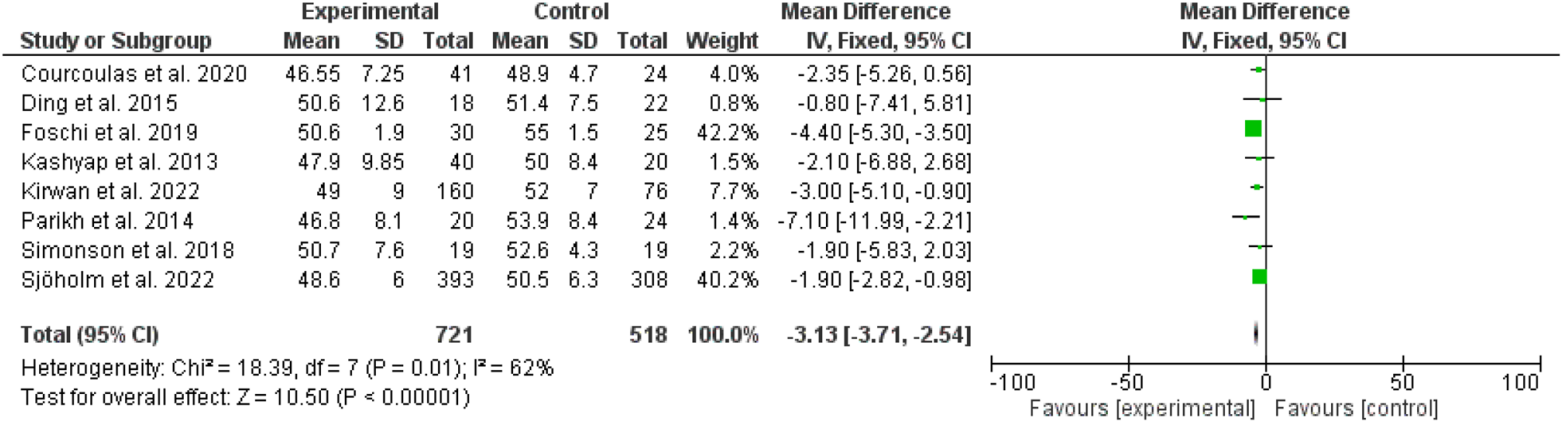
The age among different types of bariatric surgeries and medical treatments

**Fig. 5:**
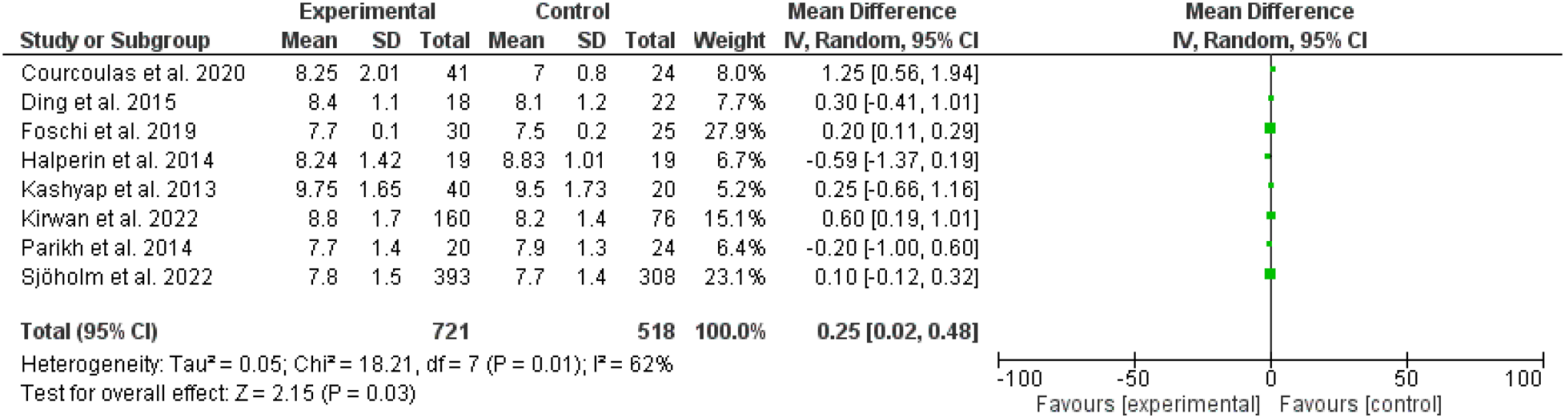
HbA1_c_ among patients with different types of bariatric surgeries and lifestyles

**Fig. 6:**
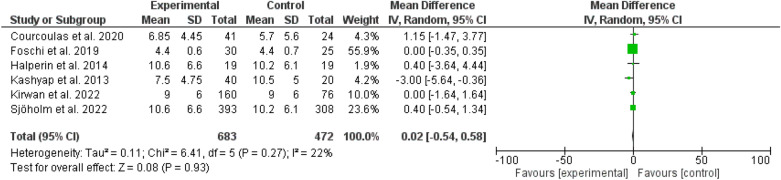
Duration of diabetes among patients with different types of bariatric surgeries and lifestyles

**Fig. 7:**
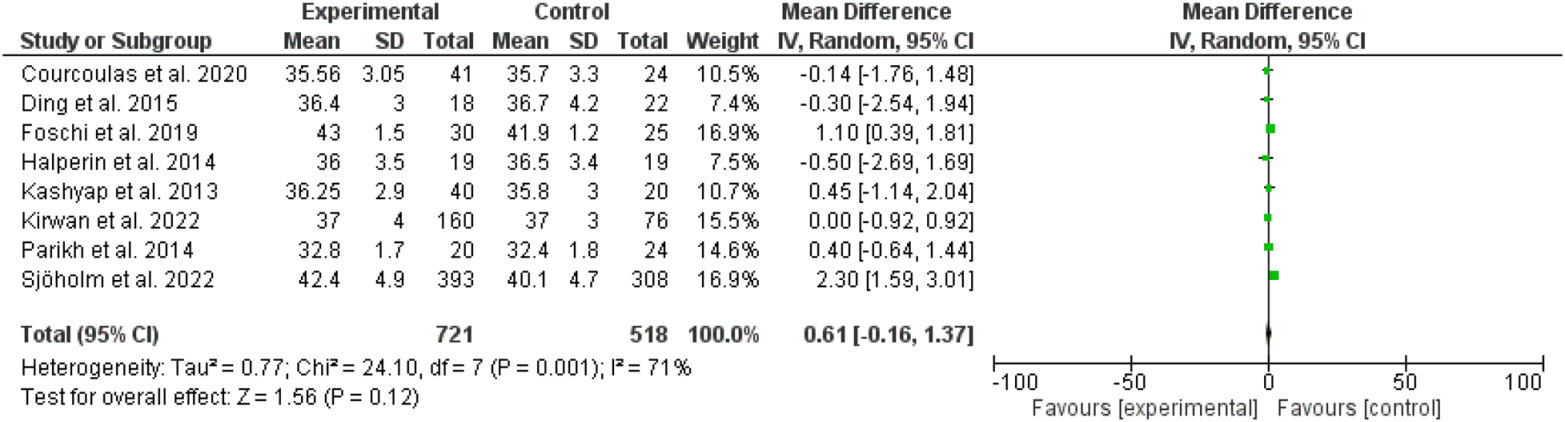
Body mass index among patients with different types of bariatric surgeries and lifestyles

**Fig. 8:**
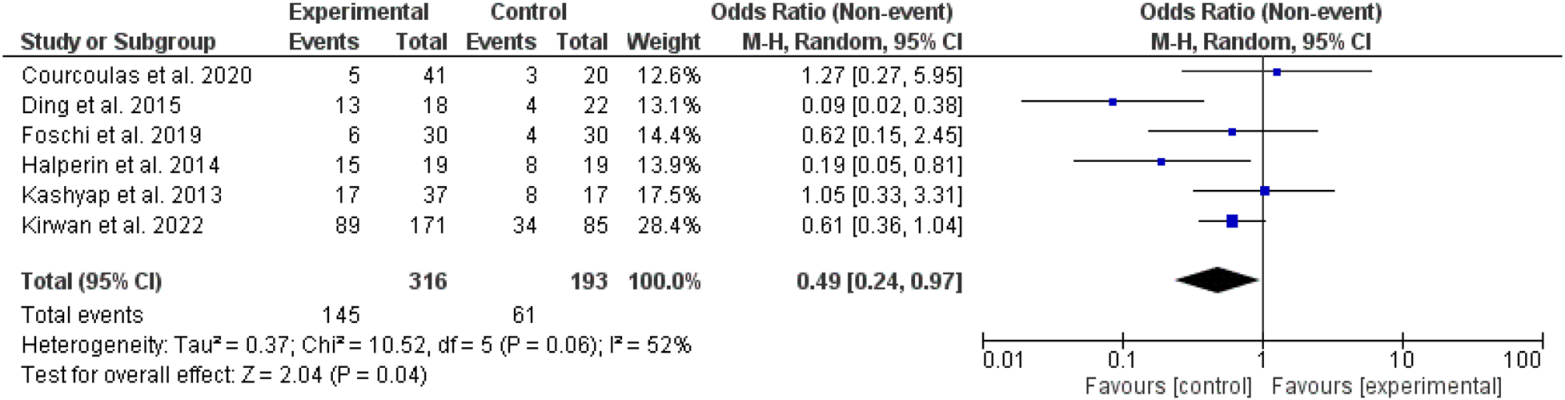
Insulin use among patients with different types of bariatric surgeries and lifestyles

**Fig. 9:**
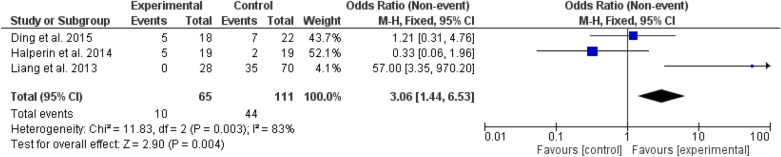
Glucagon-like peptide agonists are used among patients with different types of bariatric surgeries and lifestyles

**Fig. 10:**
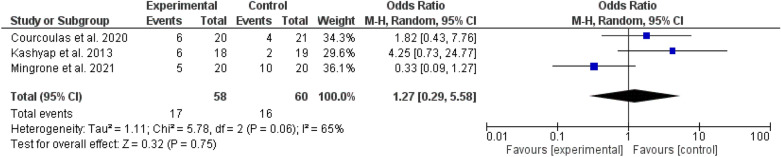
A comparison between RYGB and different types of bariatric surgeries regarding diabetes remission

A systematic literature search was conducted in PubMed MEDLINE, Web of Science, SCOPUS, and Cochrane Library from the date of the first inception up to February 2023. The reviewer searched the databases for relevant articles. The keywords diabetes remission, prolonged remission, Bariatric surgery, metabolic surgery, lifestyles, usual care, GLIP-1 agonists, insulin use, gastric banding, bypass surgery, biliopancreatic diversion, gastric bypass, sleeve gastrectomy, Roux-en-Y gastric bypass were used. In addition, the titles, abstracts, and references of the included studies were screened. We identified 432 studies and 318 stands after the removal of duplication, from them, 58 full texts were screened and only 12 studies were included in the final meta-analysis. A datasheet was used to extract the author's name year and country of publication, diabetes remission, HbA1c, age, body mass index, duration of diabetes, type of bariatric surgery, insulin, and GLP-1 agonist's use at baseline (Tables 1, 2, 3, 4, 5; Figs. 1, 2, 3, 4, 5, 6, 7, 8, 9, 10).

**Table 1:** The revised risk of bias of the included randomized controlled trials

Study	Randomization process bias	Deviation from the intended intervention	Missing outcome bias	Measurement of the outcome bias	Selective reporting results bias	Overall bias
Chong et al. 2017 [31]	High risk	Low	Unclear	Low	Low	Low
Courcoulas et al. 2020 [32]	High risk	Low	Unclear	Low	Low	Low
Ding et al. 2015 [33]	High risk	Low	Unclear	Low	Low	Low
Foschi et al. 2019 [34]	High risk	Low	Unclear	Low	Low	low
Halperin et al. 2014 [35]	High risk	Low	Unclear	Low	Low	Low
Kashyap et al. 2013 [36]	High risk	Unclear	Unclear	Unclear	Unclear	Low
Kirwan et al. 2022 [37]	High risk	Low	Unclear	Low	Low	Low
Liang et al. 2013 [38]	High risk	Low	Unclear	Low	Low	Low
Mingrone et al. 2021 [39]	High risk	Low	Unclear	Low	Low	Low
Parikh et al. 2014 [40]	High risk	Low	Unclear	Low	Low	low
Simonson et al. 2018 [41]	High risk	Low	Unclear	Low	Low	low
Sjöholm et al. 2022 [42]	High risk	Low	Unclear	Low	Low	Unclear

**Table 2:** Randomized controlled trials comparing different types of bariatric surgeries and medical treatments

Author	Country	Diabetes remission (HbA1c criteria)	Type of surgery	Patients with remission (bariatric)	Patients with remission (usual care)
Chong et al. 2017 [31]	Taiwan, USA	< 6.5	RYGB	7/36	0/35
Courcoulas et al. 2020 [32]	USA	< 6.5	RYGB, AGB	5/41	0/24
Ding et al. 2015 [33]	USA	< 6.5	AGB	5/18	2/22
Foschi et al. 2019 [34]	Italy	< 6 for complete, < 6.5 for partial	II, SG	26/30	2/25
Halperin et al. 2014 [35]	USA	< 6.5	RYGB	11/19	3/19
Kashyap et al. 2013 [36]	USA	< 6	RYGB, SG	8/40	1/20
Kirwan et al. 2022 [37]	USA	< 6.5	RYGB, SG, and AGB	60/160	2/76
Liang et al. 2013 [38]	USA	< 6.5		28/30	0/70
Mingrone et al. 2021 [39]	Italy	< 6.5	RYGB, BPD	15/40	1/20
Parikh et al. 2014 [40]	USA	< 6.5		13/20	0/24
Simonson et al. 2018 [41]	USA	< 6.5	RYGB	7/19	0/19
Sjöholm et al. 2022 [42]	Sweden	< 6.5	AGB and GB	229/393 91/393	39/308 11/308

*RYGB* Roux-en-Y gastric bypass, *AGB* adjustable gastric banding, *SG* Sleeve gastrectomy, *BPD* Biliopancreatic diversion, *II* Ileal interposition with duodenal diversion sleeve gastrectomy

**Table 3:** Basic characteristics of different types of bariatric surgeries and medical treatments

Author	Age (mean ± SD)	Sex (F/M)	BMI (kg/m^2^)	Duration diabetes	Follow-up/years
Chong et al. 2017 [31]	48.2 ± 8.4	46/71	32.4 ± 1.6	8.25 ± 5.05	Two
Courcoulas et al. 2020 [32]	46.55 ± 7.25 vs. 48.9 ± 4.7	33/41 vs. 17/20	35.56 ± 3.05 vs. 35.7 ± 3.3	6.85 ± 4.45 vs. 5.7 ± 5.6	Five
Ding et al. 2015 [33]	50.6 ± 12.6 vs. 51.4 ± 7.5	9/18 vs. 9/22	36.4 ± 3.0 vs. 36.7 ± 4.2	Not mentioned	One
Foschi et al. 2019 [34]	50.6 ± 1.9 vs. 55.0 ± 1.5	22/30 vs. 22/30	43.0 ± 1.5 vs. 41.9 ± 1.2	4.4 ± 0.6 vs. 4.4 ± 0.7	Five
Halperin et al. 2014 [35]	50.7 ± 7.6 vs. 52.6 ± 4.3	13/19 vs. 10/19	36.0 ± 3.5 vs. 36.5 ± 3.4	10.6 ± 6.6 vs. 10.2 ± 6.1	One
Kashyap et al. 2013 [36]	47.9 ± 9.85 vs. 50 ± 8.4	24/37 vs. 8/17	36.25 ± 2.9 vs. 35.8 ± 3.0	7.5 ± 4.75 vs. 10.5 ± 5.0	Two
Kirwan et al. 2022 [37]	49 ± 9 vs. 52 ± 7	137/195 vs. 75/121	37 ± 4 vs. 37 ± 3	9 ± 6 vs. 9 ± 6 STAMPEDE, TRIABETES, SLIMM-T2D, and CROSSROADS trials	Three
Mingrone et al. 2021 [39]	20/34 relapse		≥ 35	> 5 years	10 years
Parikh et al. 2014 [40]	46.8 ± 8.1 vs. 53.9 ± 8.4	23/29 vs. 22/28	32.8 ± 1.7 vs. 32.4 ± 1.8	NA	6 months
Simonson et al. 2018 [41]	50.7 ± 7.6 vs. 52.6 ± 4.3	13/19 vs. 10/19	36.3 ± 3.4. 6/19 vs. 7/19 < 35	10.6 ± 6.6 vs. 10.2 ± 6.1	Three
Sjöholm et al. 2022 [42]	48.6 ± 6.0 vs. 50.5 ± 6.3	236/393 vs. 182/308	42.4 ± 4.9 vs. 40.1 ± 4.7	NA	10 years

**Table 4:** Diabetter scoring for the included trials

Study	Diabetter score out of 9
Chong et al. 2017 [31]	8
Courcoulas et al. 2020 [32]	6
Ding et al. 2015 [33]	7
Foschi et al. 2019 [34]	6
Halperin et al. 2014 [35]	7
Kashyap et al. 2013 [36]	8
Kirwan et al. 2022 [37]	8
Liang et al. 2013 [38]	7
Mingrone et al. 2021 [39]	7
Parikh et al. 2014 [40]	7
Simonson et al. 2018 [41]	8
Sjöholm et al. 2022 [42]	6

**Table 5:** Randomized controlled trials comparing RYGB and different types of bariatric surgeries

Author	Country	Diabetes remission (HbA1c criteria)	Type of surgery	Patients with remission RYGB	Patients with remission (other surgeries)
Courcoulas et al. 2020 [32]	USA	< 6.5	RYGB, AGB	6/20	4/21
Kashyap et al. 2013 [36]	USA	< 6	RYGB, SG	6/18	2/19
Mingrone et al. 2021 [39]	Italy	< 6.5	RYGB, BPD	5/20	10/20

### Risk of bias assessment

The revised Cochrane risk of bias assessed tool (Rob.2) was used to assess the quality of the included studies [30].

### Statistical analysis

The data were analyzed by the most recent version of the RevMan system (The method used for meta-analysis was DerSimonian and Laird). We pooled 12 randomized controlled trials to assess diabetes remission, of them six studies assessed prolonged remission. The dichotomous and continuous date data were entered manually and the fixed or random effects were applied depending on heterogeneity. In addition, 8 cohorts assessed the age, body mass index, and HbA1c, between bariatric surgery and the usual care groups. In the present meta-analysis, six trials compared insulin use and diabetes duration, while three trials compared GLP-1 agonist use. A P-value of < 0.05 was considered significant.

## Results

We included 12 randomized controlled trials [31–42] and 1508 patients (846 bariatric surgery and 662 usual care including lifestyle and diabetes medications). The trials scored from 6–8 on Diabetter scoring system [43]. Diabetes remission was observed in 421 bariatric surgery patients and 193 in the usual care arm. The follow-up period ranged from 6 months to 10 years. Diabetes remission was higher among bariatric surgeries compared to usual care, odd ratio, 0.06, 95 *CI*, 0.02–0.25, Chi-square = 90.07, P-value for overall effect < 0.0001 the heterogeneity was substantial, *I*^*2*^, 88%, and P-value for heterogeneity < 0.0001 (Fig. 2).

Diabetes remission either complete or prolonged was higher among patients who underwent bariatric surgery, odd ratio, 0.12, 95 *CI*, 0.02–0.72, Chi-square = 33.63, P-value for overall effect, 0.02. The heterogeneity was significant, *I*^*2*^ for heterogeneity, 85%, and the P-value < 0.001 (Fig. 3).

Patients with bariatric surgery were younger (odd ratio, − 3.13, 95 *CI*, − 3.71 to 2.54, Chi-square = 18.39, P-value for overall effect, < 0.001, the heterogeneity was significant, *I*^*2*^, 62%, and P-value for heterogeneity, 0.01) (Fig. 4).

The glycated hemoglobin was higher among the bariatric surgery arm (odd ratio, 0.25, 95 *CI*, 0.02–0.48, Chi-square = 18.21, P-value for overall effect, 0.03 the heterogeneity was significant, *I*^*2*^, 62%, and P-value for heterogeneity, 0.01) (Fig. 5).

No differences were evident between bariatric surgery and usual care regarding the duration of diabetes (odd ratio, 0.02, 95 *CI*, − 0.54 to 0.58, P-value, 0.93), and body mass index (odd ratio, 0.61, 95 *CI*, − 0.16 to 1.37, P-value, 0.12) (Figs. 6, 7).

Insulin use was higher among the bariatric surgery arm, odd ratio, 0.49, 95% *CI*, 0.24–0.97, Chi-square, 10.52, and P-value for overall effect, 0.04, while glucagon-like agonists use was lower, odd ratio, 3.06, 95% *CI*, 1.44–6.53, Chi-square, 11.83, and P-value for overall effect, 0.004. The heterogeneity was 52% and 83% respectively (Figs 8, 9).

In the present meta-analysis, only three studies compared RYGB and other surgeries with no significant statistical difference (odd ratio, 1.27, 95% *CI,* 0.29–5.58, P-value for overall effect, 0.75, and Chi-square, 5.78. A significant heterogeneity was found, *I*^*2*^ for heterogeneity, 65%, P-value, 0.06) (Fig. 10).

## Discussion

In the present meta-analysis, bariatric surgery achieved higher diabetes remission and prolonged remission rates, odd ratio, 0.06, 95 *CI*, 0.02–0.25, and odd ratio, 0.12, 95 *CI*, 0.02–0.72); the current findings supported Schauer et al. [21]. However, Schauer and colleagues included studies with a different cut-off of diabetes remission. Cresci et al. [44] conducted a meta-analysis and found more diabetes remission among the surgery arm; their work was limited by fewer events (only six among the lifestyle group). In addition, the authors included studies with short follow-ups and studies published by the same authors. Similarly, Yu et al. [45] and Kim et al. [46] results were limited by pooling both trials and observational studies conducted among certain ethnicities. Khorgami et al. [47] included seven randomized controlled trials with short follow-up duration and found similar results. This is the largest meta-analysis updating and supporting the previous findings. We excluded studies published by the same authors and retained the most recent [32]). In addition to a sub-analysis of four randomized controlled trials [37], and a 10-year update of Mingrone et al. [39]. Furthermore, the current meta-analysis updated the previous findings and included studies with up to 10 years of follow-up. Importantly, we calculated the relapse rate (297–146 = 151 in the metabolic surgery and 186–19 = 167 in the usual care group). The results showed that 50.8% of bariatric surgery and 89.7% of the usual care relapsed with long follow-ups. The above findings imply that the majority relapsed. It is interesting to note that patients with bariatric surgery received more insulin and lower glucagon-like peptide agonists at baseline, (GLP-1 studies were not included in the complete and prolonged remission sub-analysis). Intensive Insulin therapy was found to induce diabetes remission in nearly half of the patients at one year irrespective of body weight. The effect is through the enhancement of β-cell function [48]. Anti-diabetes medications in particular sodium-glucose co-transporters-2 inhibitors and glucagon-like peptide agonists were shown to induce diabetes remission in particular when combined with other therapies including insulin [49, 50]. Metformin combination with other oral hypoglycemic medication was proven to induce diabetes remission [51, 52]. In the present study, the patient who underwent bariatric surgery were younger and had higher HbA1c (odd ratio, − 3.13, 95 CI, − 3.71 to 2.54 and 0.25, 95 CI, 0.02–0.48, respectively). No differences were evident regarding body mass index and duration of diabetes. Previous studies reported that baseline HbA1c and short duration of diabetes as predictors of diabetes remission irrespective of body mass index [53, 54]. The current findings shined the light on the complexity of diabetes mellitus and its complexity. Diabetes mellitus is a vascular and multi-system disease; it is associated with various metabolic disorders including diabetes, hypertension, dyslipidemia, and metabolic-associated fatty liver disease (MAFLD). In this view, the choice of the best intervention is based on its effects on various diabetes-associated comorbidities [55]. Weight management is crucial in holistic diabetes care [56]. However, which strategy to achieve is still to be determined. The current findings supported bariatric surgery as first-line and compared to usual care and lifestyle intervention both in complete remission and prolonged remission. However, the heterogeneity observed limited our findings (not observed in complete and prolonged remission). Bariatric surgery induces a quick surge in GLP-1 earlier in the first weeks before weight loss [57]. The novel anti-diabetic medications including GLP-1-like agonists were recently approved for both diabetes and weight management. In addition, diabetes remission was observed in 66% to 81% [57]. Moreover, GLP-1 agonists reduced MAFLD, cardiac remodeling, and reduced pancreatic fat [50, 58, 59]. The most important question is who benefits the most? What is the role of novel antidiabetic medications? In addition, which intervention is for a particular patient with particular comorbidities? The strength of the current meta-analysis was the use of the recent definition of diabetes remission, the assessment of prolonged remission and relapse rate, and the assessment of insulin and GLIP-1 agonists use. In addition, we excluded studies published by the same authors and included new updates with longer follow-ups.

### A comparison of different bariatric surgeries regarding diabetes remission

All types of bariatric surgery were superior to usual care in diabetes remission. The current study pooled various types of bariatric surgery, which is a major limitation. However, Uhe et al. [60] compared Roux-en-Y gastric bypass, sleeve gastrectomy, or one-anastomosis gastric bypass and found no difference regarding diabetes remission in agreement with the current findings in which there was no significant difference between RYGB and other types of bariatric surgeries. The small number of the included studies limited Uhe and colleagues' meta-analysis (three, six, and three at 3 months, one year, and 5 years, respectively).

Ding et al. [61] compared usual care and six bariatric surgeries and found that all were superior to usual care with Mini-gastric bypass the better, followed by biliopancreatic diversion, laparoscopic sleeve gastrectomy, and Roux-en-Y gastric bypass. However, at three years BPD, and mini-GBP were better. When considering all obesity comorbidities, Roux-en-Y gastric bypass was the best choice. In this meta-analysis, it is not possible to compare all six types of bariatric procedures.

Castellana et al. [62] included ten randomized controlled trials and compared the most two commonly used bariatric surgeries and found the superiority of Roux-en-Y gastric bypass over laparoscopic sleeve gastrectomy in the short-term. A recent interesting meta-analysis conducted by Fehervari et al. [63] found good weight loss and diabetes remission after sleeve gastrectomy conversion to Roux-en-Y gastric bypass. In this meta-analysis we compared RYGB arm with gastric banding, sleeve gastrectomy, and biliopancreatic diversion and no difference was found. A plausible explanation is the small number of the studies included. In addition, we compared malabsorption procedure in one arm with restrictive and malabsorption in the other arm.

Kwon et al. [64] assessed the length of the biliopancreatic and Roux limb in Roux-en-Y gastric bypass and found a higher rate of diabetes remission in the longer biliopancreatic but not the Roux limb. A more recent meta-analysis published in France [65] found that all bariatric surgeries were superior to medical treatment with one anastomosis gastric bypass and biliopancreatic diversion being the most effective followed by Roux-en-Y gastric bypass. Importantly, the authors observed a progressive decrease in diabetes remission over time regardless of the intervention. Li et al. [66] compared metabolic surgeries, restrictive procedures, and lifestyles and found that bariatric surgery was more effective in diabetes remission.

In conclusion, all types of bariatric surgeries were superior to lifestyle and usual care with mini-gastric bypass being the best followed by biliopancreatic diversion. Roux-en-Y gastric bypass was the best when considering all obesity comorbidities especially if the biliopancreatic limb is long. RGYB might be better in the real world because diabetes mellitus is usually accompanied by other comorbidities including hypertension dyslipidemia, and gastroesophageal reflux [67–69]. The high relapse needs to be observed in different interventions calls for longer randomized controlled trials.

The study limitations were the pooling of different bariatric surgeries, the high heterogeneity observed in some analyses, no information available regarding the level of exercise among the bariatric surgery patients, and the fact that we could not control for the different oral hypoglycemic medications.

## Conclusion

Bariatric surgery was better than usual care in diabetes remission both in the short and long term. Bariatric surgery patients were younger, had higher HbA1c, and received more insulin and lower GLP-1-like agonists. No differences were evident regarding body mass index and the duration since diabetes diagnosis. No difference was found between RYGB and other bariatric surgeries regarding diabetes remission. Further trials comparing the new anti-diabetic medications and different forms of bariatric surgery and controlling for the level of exercise and diet are needed.

## Data Availability

The data used in this manuscript are available upon request.

## Abbreviations

RYGB: Roux-en-Y gastric bypass
AGB: Adjustable gastric banding
SG: Sleeve gastrectomy
BPD: Biliopancreatic diversion
II: Ileal interposition with duodenal diversion sleeve gastrectomy
GLP-1 agonists: Glucagon-like peptide-1 agonists
